# Adjuvant potential of *Satureja hortensis* metabolites with antibiotics against Gram-positive and Gram-negative bacterial strains

**DOI:** 10.1080/13880209.2025.2572669

**Published:** 2025-10-15

**Authors:** Marah Alburqan, Annamária Kincses, Melinda Paizs, Anita Barta, Katalin Veres, Antal Csámpai, Morteza Yazdani, Judit Hohmann

**Affiliations:** a Institute of Pharmacognosy, University of Szeged, Szeged, Hungary; b Department of Organic Chemistry, Eötvös Loránd University, Budapest, Hungary; c HUN-REN – USZ Biologically Active Natural Products Research Group, University of Szeged, Szeged, Hungary

**Keywords:** Flavones, naringenin, rosmarinic acid, cilicione-a, fulgidic acid, combinations with antibiotics, adjuvant

## Abstract

**Context:**

The global rise in bacterial resistance to existing antibiotics has led to increased mortality rates and escalating healthcare costs, underscoring the urgent need for new classes of antibacterial agents that can act synergistically with current antimicrobials.

**Objective:**

This study aimed to isolate bioactive compounds from *Satureja hortensis* L. (Lamiaceae) and evaluate their effects in combination with ciprofloxacin (CIP), ampicillin (AMP), gentamicin (GEN), and tetracycline (TET).

**Materials and methods:**

The adjuvant potential of naringenin (N), 5,6-dihydroxy-7,3′,4′-trimethoxyflavone (TMF), cilicione-a (Cil), rosmarinic acid (Rs), rosmarinic acid methyl ester (MRs), and fulgidic acid was evaluated by determining the minimum inhibitory concentrations (MICs) of selected antibiotics in presence of sub-inhibitory concentrations of these compounds. The assays were performed against ten bacterial strains using the twofold broth microdilution method. Test samples were prepared in 96-well microtiter plates, incubated at 37 °C for 18 h, and dimethyl sulfoxide was used as the solvent control.

**Results:**

N, Rs, and MRs reduced the MIC value of GEN from 0.78 µM to 0.097 µM against the drug-resistant *Escherichia coli* AG100 strain. Additionally, the combinations of N and MRs with CIP reduced its MIC from 3.125 µM to 0.39 µM against *Klebsiella pneumoniae*. A MIC reduction (from 50 µM to 12.5 µM) was observed against the methicillin- and oxacillin-resistant *Staphylococcus aureus* strain when N, Cil, Rs, and MRs were combined with AMP. The MIC changed from 0.125 µM to 0.03125 µM against *Bacillus subtilis* when N and TMF were combined with GEN.

**Discussion and conclusions:**

These findings highlight the potential of such combinations to enhance antibiotic efficacy, lower the required dosages, and contribute to mitigating the emergence of antimicrobial resistance.

## Introduction

The rise and global dissemination of multidrug-resistant (MDR) bacteria pose a major public health challenge, significantly compromising the efficacy of current treatments for infectious diseases. According to the World Health Organization (WHO), the progressive reduction in therapeutic options—primarily driven by bacterial resistance—has led to increased morbidity and mortality associated with bacterial infections (Matsunaga and Hayakawa 2018; Getahun et al. 2022). In response, intensified research aimed at identifying novel antimicrobial agents, optimizing existing therapeutic combinations, and elucidating synergistic interactions is essential for developing effective antibacterial strategies.

Synergistic combinations with antibiotics exert effects greater than the sum of their individual actions, enhancing efficacy while reducing required doses and minimizing side effects. The diminished effectiveness of antibiotics is influenced not only by microbial resistance but also by host-related factors such as immunodeficiency, advanced age, transplantation, and physiological stress. Recent studies have highlighted the potential of synergistic interactions between antibiotics and herbal compounds or phytochemicals. Co-administration of phytochemicals with antibiotics has been shown to enhance antibacterial efficacy and suppress the emergence of resistance (Ayaz et al. 2019). Additionally, antibiotic adjuvants can potentiate antibacterial activity by directly targeting bacterial mechanisms, bypassing intrinsic resistance, or improving antibiotic performance within the host (Dhanda et al. 2023).

The genus *Satureja* L. (savory) comprises approximately 200 aromatic herbs and shrubs with global distribution. Species within this genus are used in traditional and modern medicine, as well as in culinary and agricultural applications. Traditionally, *Satureja* species have been employed to treat respiratory and gastrointestinal disorders and are recognized for their antifungal and antibacterial properties. *Satureja hortensis* L. (summer savory) (Lamiaceae), an annual species native to southeastern Europe and western Asia, is widely cultivated as a culinary herb. Its extracts have demonstrated notable antioxidant, cytotoxic, and antibacterial activities in various bioassays (Ejaz et al. 2023).

This study aimed to identify the bioactive constituents of *S. hortensis* and evaluate their adjuvant potential in combination with various antibiotics. The antibacterial activities of ten compounds (**1**–**10**), isolated from the methanolic extract of the aerial parts, were assessed individually and in combination with conventional antibiotics against clinically relevant Gram-positive and Gram-negative bacterial strains.

## Materials and methods

### Experimental procedures of compound isolation and structure determination

Flash chromatography (FC) was performed on a CombiFlash Rf + Lumen instrument with integrated UV, UV–Vis, and ELS detection using a reversed-phase (RP) flash column (RediSep C_18_ Bulk 950, Teledyne Isco, Lincoln, NE, USA, Product No. Lab-Ex Kft. 692203336). RP high-performance liquid chromatography (RP-HPLC) and normal-phase HPLC (NP-HPLC) separations were performed on a Shimadzu LC-10 A S HPLC instrument equipped with a UV–Vis detector (Shimadzu, Co., Ltd., Kyoto, Japan) using RP-HPLC (LiChrospher RP-18, 5 μm, 250 × 4 mm, Merck, Darmstadt, Germany, Product No. Lab-Ex Kft. 00 G-3050-D0) and NP-HPLC (LiChrospher Si 60, 5 μm, 250 × 4 mm, Merck, Product No. Gen-Lab Kft., 00 G-3047-D0) columns, respectively. Sephadex LH-20 (25–100 μm, Cytiva, Sweden AB, Uppsala, Sweden, Product No. Merck GE17-0090-10) was used for gel filtration. Vacuum liquid chromatography (VLC) was performed on silica gel (GF_254_, 15 µm, Product No. Merck, 1.07730). Thin-layer chromatography (TLC) was performed using silica plates (20 × 20 cm silica gel 60 F_254_, Product No. Merck 1055540001). Analytical grade solvents were supplied by Molar Chemicals Kft. (Halásztelek, Hungary): methanol (Product No. 05730-006-410), *n*-hexane (Product No. 06850-101-411), cyclohexane (Product No. 01960-101-340), chloroform (Product No. 04730-101-411), ethyl acetate (Product No. 02930-101-411), etanol (Product No. 02911-101-340), and acetonitril (Product No. 06850-101-411). Solvents for HPLC were purchased from VWR International Kft. (Debrecen, Hungary): ethyl acetate (Product No. 83621.320), cyclohexane (Product No. 83629.320), methanol (Product No. 20864.320), and acetonitril (Product No. 20060.320). The water was supplied by the Milli-Q Direct 3 UV pump (Merck, Darmstadt, Germany). The structures were determined by one-dimensional (1D) (^1^H NMR,^13^C NMR JMOD) and two-dimensional (2D) NMR (^1^H-^1^H correlation spectroscopy [COSY], nuclear overhauser effect spectroscopy, heteronuclear single quantum coherence [HSQC], and heteronuclear multiple bond correlation [HMBC]) spectroscopy. NMR spectra were recorded on a Bruker Avance DRX 500 spectrometer (Bruker, Billerica, MA, USA) at 500 MHz (^1^H) and 125 MHz (^13^C). The signals of the deuterated solvents were taken as references. 2D NMR measurements were performed with standard Bruker software. In the ^1^H-^1^H COSY, HSQC, and HMBC experiments, gradient-enhanced versions were applied.

### Plant material

Aerial parts of *S. hortensis* L. (Lamiaceae) were collected in the flowering stage by the grower Ferenc Okvátovity in Bátya, Hungary, in August 2022. A voucher specimen (Voucher No. 948) was deposited at the Herbarium of Department of Pharmacognosy, University of Szeged, Hungary. The plant material was evenly spread in a thin layer and air-dried at room temperature under protected conditions.

### Extraction and isolation

The dried aerial parts of *S. hortensis* (1.9 kg) were exhaustively percolated with methanol (12 L) at room temperature. The resulting extract was concentrated under reduced pressure, dissolved in 50% aqueous methanol, and subjected to solvent–solvent partitioning with *n*-hexane (10 × 600 mL), chloroform (10 × 600 mL), and ethyl acetate (10 × 600 mL).

The chloroform-soluble fraction (8.27 g) was subjected to open-column chromatography on polyamide (150 g) and eluted with a stepwise methanol–water gradient (1:4, 2:3, 3:2, 4:1, v/v) followed by 100% methanol, yielding fractions A–E corresponding to 20%, 40%, 60%, 80%, and 100% methanol elutions, respectively. Fraction B (1.28 g), eluted with 40% methanol, was further purified by reversed-phase FC (RP-FC) on RP-silica gel (24 g) using a linear gradient of water–methanol (0% to 100%) over 75 min. Fractions with similar TLC profiles (B1–B22) were combined. Crystallization of fraction B15 yielded pure fulgidic acid (**8**) (115.6 mg). Fraction C (1.92 g), eluted with 60% methanol, was subjected to RP-FC under the same conditions (RP-silica gel, 24 g; water–methanol gradient, 0% to 100%) over 70 min, affording fractions C1–C16 based on TLC monitoring. From fraction C6, compound **4**—identified as 5,6-dihydroxy-7,3′,4′-trimethoxyflavone (**4**) (113.5 mg)—was crystallized using methanol as the solvent. Fraction D (1.06 g), eluted with 80% methanol, was separated by RP-FC on RP-silica gel (24 g) using a linear water–methanol gradient (20% to 100%) over 70 min, yielding subfractions D1–D10. Subfraction D2 was further purified by Sephadex LH-20 gel filtration with methanol as the eluent, affording five subfractions (D2/1–D2/5). Subfraction D2/4 was further purified by preparative TLC on silica gel plates using chloroform–methanol (97:3, v/v) as the developing solvent, followed by NP-HPLC using an isocratic ethyl acetate–cyclohexane (70:30, v/v) solvent system, yielding naringenin (**1**) (1.62 mg). From subfraction D2/5, a crystalline material identified as 5,6,4′-trihydroxy-7,3′-dimethoxyflavone (**3**) (1.3 mg) was obtained.

The ethyl acetate-soluble fraction (20.5 g) was subjected to normal-phase VLC (NP-VLC) on silica gel using a gradient system of cyclohexane–ethyl acetate–ethanol (60:15:0, 60:20:0.5, 60:30:1, 60:30:3, 60:30:5, 60:30:10, 60:30:20, and 50:50:30, v/v/v), followed by ethyl acetate–methanol (8:2, v/v). Fractions were combined based on TLC monitoring to yield 13 pooled fractions (F1–F13). Fraction F3 (159.31 mg) was purified by RP-FC on RP-silica gel (40 g) using a linear water–acetonitrile gradient (0% to 100%) over 60 min, affording subfractions F3/1–F3/15. Crystallization of subfraction F3/13 from methanol yielded a crystalline compound identified as cilicione-a (**5**) (3.88 mg). Compound **2** (2.21 mg), identified as apigenin, was isolated from subfraction F3/15 by repeated RP-FC. Fraction F4 (799.21 mg) was further separated by RP-FC on RP-silica gel (40 g) using a linear water–methanol gradient (0% to 100%) over 70 min, yielding subfractions F4/1–F4/15. Subfraction F4/3 (67.37 mg) was further purified by gel filtration on Sephadex LH-20 using methanol as the eluent, followed by RP-HPLC with an isocratic water–methanol system (70:30, v/v), yielding 3-hydroxytyrosol (**10**) (3.69 mg). Fraction F6 (3.075 g) was purified by RP-FC on RP-silica gel (40 g) using a linear water–acetonitrile gradient (0% to 100%) over 75 min, affording subfractions F6/1–F6/21. Subfraction F6/15 (273.47 mg) was further separated by RP-FC using a similar gradient (0% to 100% acetonitrile in water over 70 min), yielding subfractions F6/15.1–F6/15.13. Subfraction F6/15.7 was purified by RP-HPLC with an isocratic acetonitrile–water system (1:1, v/v), affording carvacrol-*β*-*O*-glucoside (**9**) (1.46 mg). Subfraction F6/15.9 (133 mg) was subjected to RP-VLC on C_18_ silica gel using a water–acetonitrile gradient (10% to 50%), yielding rosmarinic acid methyl ester (**7**) (6.65 mg). Furthermore, subfraction F6/18 (38.91 mg) was purified by RP-VLC using a water–acetonitrile gradient (0% to 50%) to yield rosmarinic acid (**6**) (7.33 mg).

*5,6,4′-Trihydroxy-7,3′-dimethoxyflavone* (**3**): pale yellow crystals; Mp. 268 °C–271 °C; ^1^H NMR (500 MHz, CD_3_OD, *δ* ppm): 7.53 (1H, dd, *J* = 8.2, 1.7 Hz, H-6′), 7.50 (1H, d, *J* = 1.8 Hz, H-2′), 6.92 (1H, d, *J* = 8.3, H-5′), 6.84 (1H, *s*, H-8), 6.64 (1H, *s*, H-3), 3.99 (3H, *s*, 7-OCH_3_), and 3.97 (3H, *s*, 3′-OCH_3_); ^13^C NMR (125 MHz, CD_3_OD, *δ* ppm): 184.3 (C-4), 166.5 (C-2), 155.8 (C-7), 152.8 (C-4′), 152.0 (C-9), 149.7 (C-3′), 147.2 (C-5), 131.5 (C-6), 123.4 (C-1′), 121.8 (C-6′), 117.0 (C-5′), 110.6 (C-2′), 106.6 (C-10), 103.7 (C-3), 91.9 (C-8), 57.0 (7-OCH_3_), and 56.7 (3′-OCH_3_).

*Cilicione-a* (*5*): yellow solid; ^1^H NMR (500 MHz, CD_3_OD, *δ* ppm): 7.35 (2H, d, *J* = 8.2 Hz, H-2, H-6), 6.83 (2H, *J* = 8.2 Hz, H-3, H-5), 5.93 (1H, *s*, H-8), 5.89 (1H, *s*, H-3′), 4.98 (1H, d, *J* = 11.6 Hz, H-7), 4.54 (1H, d, *J* = 11.6 Hz); ^13^C NMR (125 MHz, CD_3_OD, *δ* ppm): 198.5 (C-9), 168.9 (C-4′), 165.3 (C-6′), 164.6 (C-2′), 159.2 (C-4), 130.4 (C-2, C-6), 129.3 (C-1), 116.2 (C-3, C-5), 101.8 (C-1′), 97.4 (C-5′), 96.4 (C-3′), 85.0 (C-7), 73.7 (C-8).

*Fulgidic acid* (**8**): white amorphous solid; ^1^H NMR (500 MHz, DMSO-*d*_6_, *δ* ppm): 5.58 (2H, m, H-10, H-11), 5.40 (1H, dt, *J* = 6.8, 10.9 Hz, H-15), 5.36 (1H, dt, *J* = 6.8, 10.9 Hz, H-16), 3.90 (1H, brs, H-9), 3.82 (1H, *m*, H-12), 3.28 (1H, *m*, H-13), 2.19 (1H, *m*, H-14a), 2.17 (2H, *t*, *J* = 7.4 Hz, H-2), 1.97 (2H, *t*, *J* = 7.5 Hz, H-17), 1.91 (1H, *m*, H-14b), 1.47 (2H, *m*, H-3), 1.36 (2H, *m*, H-8), 1.24 (8H, *m*, H-4–H-7), and 0.97 (3H, *t*, *J* = 7.5 Hz, H-18); ^13^C NMR (125 MHz, DMSO-*d*_6_, *δ* ppm): 174.5 (C-1), 134.6 (C-10), 131.9 (C-16), 129.3 (C-11), 126.6 (C-15), 74.0 (C-13), 73.6 (C-12), 70.5 (C-9), 37.4 (C-8), 33.7 (C-2), 30.0 (C-14), 29.0, 28.8, 28.5, 24.9 (C-4–C-7), 24.5 (C-3), 20.2 (C-17), and 14.1 (C-18).

*Carvacrol-β-O-glucoside* (**9**): colorless oil; ^1^H NMR (500 MHz, CD_3_OD, *δ* ppm): 7.02 (1H, d, *J* = 8.0 Hz, H-3), 7.01 (1H, brs, H-6), 6.78 (1H, brd, *J* = 7.7 Hz, H-4), 4.86 (1H, H-2′), d, *J* = 7.1 Hz, H-1′), 3.89 (1H, d, *J* = 11.9 Hz, H-6’a), 3.70 (1H, dd, *J* = 5.1, 11.9 Hz, H-6’b), 3.48 (1H, *m*, H-2′), 3.47 (1H, *m*, H-3′), 3.41 (1H, *m*, H-5′), 3.40 (1H, *m*, H-4′), 2.84 (1H, sept, *J* = 6.6 Hz), 2.22 (3H, *s*, H-7), and 1.22 (6H, d, *J* = 6.6 Hz, H-9, H-10); ^13^C NMR (125 MHz, CD_3_OD, *δ* ppm): 157.2 (C-1), 149.1 (C-5), 131.4 (C-3), 126.2 (C-2), 121.2 (4), 114.6 (C-6), 102.9 (C-1′), 78.3 (C-3′), 78.2 (C-5′), 75.1 (C-2′), 71.6 (C-4′), 62.7 (C-6′), 35.2 (C-8), 24.5 (C-10), 24.4 (C-9), and 16.1 (C-7). NMR data are in agreement with the literature values (Mastelić et al., 2004).

### Comparative density functional theory (DFT) modeling study of cilicione-a (5)

Calculations were carried out with M062X global functional (Zhao and Truhlar 2008) using 6–31 G(d,p) basis set (Hehre et al. 1986) and supplemented by the integral equation formalism polarizable continuum solvent model (Tomasi et al. 1999). The diagnostic theoretical coupling constants were calculated using the online generalized ^3^*J*_HH_ calculation program elaborated by Haasnoot et al. (1980).

### Bacterial strains

In this study, the following Gram-positive bacterial strains were examined: *Staphylococcus aureus* ATCC 29213; methicillin- and oxacillin-resistant *S. aureus* (MRSA) ATCC 43300; *Staphylococcus epidermidis* ATCC 12228; *Enterococcus faecalis* ATCC 29212; and *Bacillus subtilis* ATCC 6633. The Gram-negative bacterial strains tested included *Escherichia coli* ATCC 35218; *E. coli* K-12 AG100, which expresses the AcrAB-TolC efflux pump at basal levels; *Salmonella enterica* serovar Typhimurium SL1344 (SEO1); *Klebsiella pneumoniae* ATCC 700603; and *Pseudomonas aeruginosa* ATCC 27853. The *Salmonella* strain was kindly provided by Dr. Jessica M. A. Blair (University of Birmingham, UK).

### Determination of minimum inhibitory concentrations (MICs)

The MICs of all tested compounds and antibiotics were determined according to the guidelines of the Clinical and Laboratory Standards Institute (CLSI 2018). MICs were established using twofold serial dilutions of the compounds (200–0.0195 µM) in Mueller–Hinton Broth (MHB, Biolab Zrt., Product No. MHC20500), with a standardized bacterial inoculum of 5 × 10^5^ CFU/mL. The turbidity of the bacterial suspension was adjusted using a McFarland densitometer (Biosan, Riga, Latvia). Assays were performed in 96-well microtiter plates and incubated at 37 °C for 18 h. MICs were determined visually at the end of the incubation period. Dimethyl sulfoxide (DMSO, Molar Chemicals Kft., Product No. 02610-526-340) served as the solvent control, with its final concentration (2% v/v) verified to have no antibacterial activity. Results are presented as mean values from three replicates, each conducted in three independent experiments. The reference antibiotics—ciprofloxacin (CIP) (Product No. Merck 17850-5 G-F**)**, ampicillin sodium crystalline (AMP) (Sigma Aldrich, Product No. A9518-5G), gentamicin sulfate (GEN) (Sigma Aldrich, Product No. G1264-250), and tetracycline hydrochloride (TET) (Sigma Aldrich, Product No. T7660-5G)—were used.

### Enhancement of the activity of antibiotics

The chemosensitizing potential of compounds **1** and **4–8** was evaluated by determining the MIC values of selected antibiotics in the presence of fixed sub-inhibitory concentrations (100 µM) of each compound against Gram-positive and Gram-negative bacterial strains. MICs were determined using the standard twofold broth microdilution method in 96-well microtiter plates, with serial dilutions of CIP, AMP, GEN, and TET. In each assay, the first four rows of the plate contained twofold serial dilutions of the antibiotics alone, while the last four rows assessed the antibiotic–compound combinations. A 10^−4^ dilution of overnight bacterial cultures in 50 µL of MHB was added to each well, except for medium control wells. Plates were incubated at 37 °C for 18 h, and MICs were determined visually at the end of the incubation period. All results are reported as mean values from three biological replicates, each conducted in three independent experiments.

## Results

**Figure 1.**
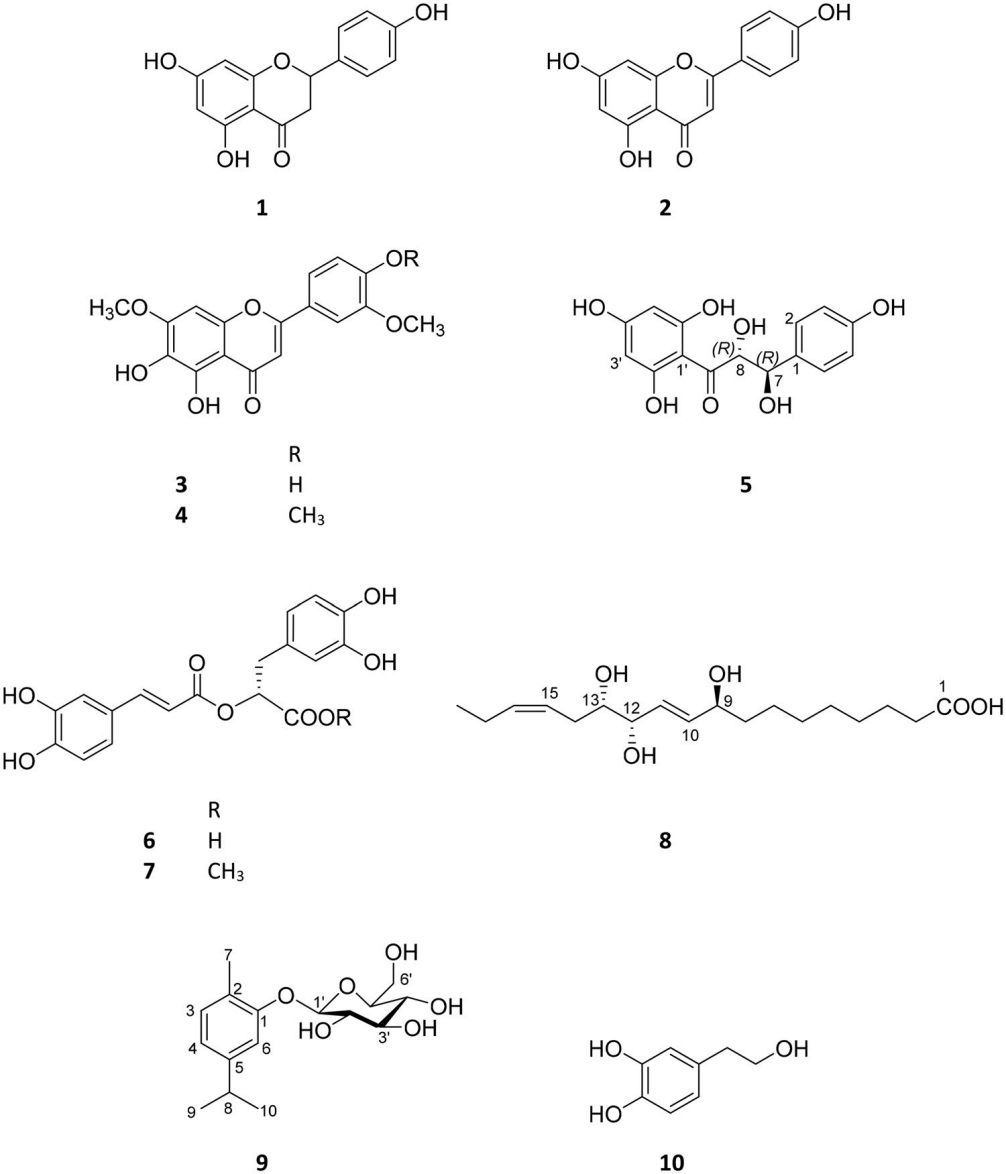
Structure of the investigated compounds: naringenin (**1**), apigenin (**2**), 5,6,4′-trihydroxy-7,3′-dimethoxyflavone (**3**), 5,6-dihydroxy-7,3′,4′-trimethoxyflavone (**4**), cilicione-a (**5**), rosmarinic acid (**6**), rosmarinic acid methyl ester (**7**), fulgidic acid (**8**), carvacrol-*β*-*O*-glucoside (**9**), and 3-hydroxytyrosol (**10**).

In this study, the aerial parts of *S. hortensis* were subjected to detailed phytochemical investigation. Ten compounds (**1**–**10**) were isolated from the chloroform- and ethyl acetate-soluble phases of the methanolic extract using a multistep chromatographic purification procedure. The structures of the isolated compounds were elucidated by spectroscopic methods and identified as naringenin (**1**), apigenin (**2**), 5,6,4′-trihydroxy-7,3′-dimethoxyflavone (**3**), 5,6-dihydroxy-7,3′,4′-trimethoxyflavone (**4**), cilicione-a (**5**), rosmarinic acid (**6**), rosmarinic acid methyl ester (**7**), fulgidic acid (**8**) [= (9*S*,12*S*,13*S*)-9,12,13-trihydroxy-10*E*,15*Z*-octadecadienoate], carvacrol-*β*-*O*-glucoside (**9**), and 3-hydroxytyrosol (**10**) (Figure 1).

Compounds **1** and **4**–**8**, in sufficient amounts, in combination with antibiotics were investigated for their possible adjuvant effects. In this study, antibiotics representing different structural classes were selected: CIP (fluoroquinolone class), GEN (aminoglycoside), AMP (β-lactam), and TET (tetracyclic derivative). These antibiotics exhibit distinct mechanisms of action, targeting the bacterial cell wall (AMP), nucleic acids (CIP), or protein biosynthesis (GEN and TET). First, the MICs were determined for **1** and **4**–**8** (Table 1(A)) and for the antibiotics (Table 1(B)) against five Gram-positive and five Gram-negative bacterial strains. The MICs of all compounds were >200 µM, whereas most of the bacterial strains used in the study were sensitive to antibiotics, with some exceptions (MIC >100 µM) presented in Table 1(A).

**Table 1A.** MIC values (µM) of the tested compounds **1**, **4–8** and antibiotics.

Bacterial strains		MIC (µM)					MIC (v/v %)
		1, 4–8	CIP	AMP	GEN	TET	DMSO
Gram-positive bacteria	*S. aureus* ATCC 29213	>200	1.25	5	1.25	2.5	>1
	*S. aureus* MRSA ATCC 43300	>200	1.25	50	100	2.5	>1
	*S. epidermidis* ATCC 12228	>200	0.39	3.125	0.0625	>100	>1
	*E. faecalis* ATCC 29212	>200	1.56	6.25	25	100	>1
	*B. subtilis* ATCC 6633	>200	0.125	0.5	0.125	0.625	>1
Gram-negative bacteria	*E. coli* ATCC 35218	>200	0.039	>100	1.25	6.25	>1
	*E. coli* AG100	>200	0.049	25	0.78	6.25	>1
	*S.* Typhimurium SEO1	>200	0.0156	6.25	0.78	6.25	>1
	*K. pneumoniae* ATCC 700603	>200	3.125	>100	12.5	>100	>1
	*P. aeruginosa* ATCC 27853	>200	0.625	>100	1.25	>100	>1

The MICs of antibiotics were subsequently determined in the presence of a sub-inhibitory concentration (100 µM) of each compound in both Gram-positive and Gram-negative bacterial strains to evaluate their chemosensitizing potential. The potentiating effects of the antibiotics and *S. hortensis* metabolites were assessed using a twofold broth microdilution method in 96-well plates. The results are presented in Tables 2–5.

**Table 1B.** MIC values of the antibiotics in µg/mL.

Bacterial strains		MIC (µg/mL)			
		CIP	AMP	GEN	TET
Gram-positive bacteria	*S. aureus* ATCC 29213	0.414	1.857	1.861	1.202
	*S. aureus* MRSA ATCC 43300	0.414	18.570	148.88	1.202
	*S. epidermidis* ATCC 12228	0.129	1.161	0.093	>48.09
	*E. faecalis* ATCC 29212	0.517	2.321	37.22	48.09
	*B. subtilis* ATCC 6633	0.041	0.186	0.186	0.301
Gram-negative bacteria	*E. coli* ATCC 35218	0.012	>37.139	1.861	3.006
	*E. coli* AG100	0.016	9.285	1.163	3.006
	*S.* Typhimurium SEO1	0.005	2.321	1.163	3.006
	*K. pneumoniae* ATCC 700603	1.035	>37.139	18.61	>48.09
	*P. aeruginosa* ATCC 27853	0.207	>37.139	18.61	>48.09

**Table 2.** Reduction of MIC values of ciprofloxacin (CIP) in combination with compounds **1**, **4–8** in bacterial strains*.

Bacterial strains		MIC (µM)						
		CIP	CIP +					
			1	4	5	6	7	8
Gram-positive bacteria	*S. aureus* ATCC 29213	1.25	**2-fold**	None	None	None	None	None
	*S. aureus* MRSA ATCC 43300	1.25	**2-fold**	None	None	**2-fold**	**2-fold**	None
	*S. epidermidis* ATCC 12228	0.39	None	None	None	None	None	None
	*E. faecalis* ATCC 29212	1.56	None	None	ND	None	**2-fold**	None
	*B. subtilis* ATCC 6633	0.125	**2-fold**	**2-fold**	**2-fold**	**2-fold**	**2-fold**	**2-fold**
Gram-negative bacteria	*E. coli* ATCC 35218	0.039	2-fold increase	None	ND	None	None	None
	*E. coli* AG100	0.049	None	None	ND	None	2-fold increase	None
	*S.* Typhimurium SEO1	0.0156	2-fold increase	2-fold increase	None	2-fold increase	2-fold increase	2-fold increase
	*K. pneumoniae* ATCC 700603	3.125	**8-fold**	2-fold increase	**4-fold**	**4-fold**	**8-fold**	2-fold increase
	*P. aeruginosa* ATCC 27853	0.625	**2-fold**	2-fold increase	**2-fold**	**2-fold**	**2-fold**	2-fold increase

*The bold letters indicate a decrease in the MIC of CIP; ND: not determined.

**Table 3.** Reduction of MIC values of gentamicin (GEN) in combination with compounds **1**, **4–8** in bacterial strains*.

Bacterial strains		MIC (µM)						
		GEN	GEN +					
			1	4	5	6	7	8
Gram-positive bacteria	*S. aureus* ATCC 29213	1.25	**4-fold**	**2-fold**	None	None	None	**2-fold**
	*S. aureus* MRSA ATCC 43300	100	**4-fold**	None	**2-fold**	**2-fold**	**2-fold**	None
	*S. epidermidis* ATCC 12228	0.0625	None	None	ND	None	None	None
	*E. faecalis* ATCC 29212	25	**2-fold**	None	ND	**2-fold**	**2-fold**	None
	*B. subtilis* ATCC 6633	0.125	**4-fold**	**4-fold**	**2-fold**	**2-fold**	**2-fold**	**2-fold**
Gram-negative bacteria	*E. coli* ATCC 35218	1.25	2-fold increase	None	ND	None	2-fold increase	None
	*E. coli* AG100	0.78	**8-fold**	**2-fold**	ND	**8-fold**	**8-fold**	None
	*S.* Typhimurium SEO1	0.78	2-fold increase	None	2-fold increase	2-fold increase	2-fold increase	None
	*K. pneumoniae* ATCC 700603	12.5	None	None	None	2-fold increase	2-fold increase	**2-fold**
	*P. aeruginosa* ATCC 27853	1.25	None	None	None	None	None	None

*The bold letters indicate a decrease in the MIC of GEN; ND: not determined.

**Table 4.** Reduction of MIC values of ampicillin (AMP) in combination with compounds **1**, **4–8** in bacterial strains*.

Bacterial strains		MIC (µM)						
		AMP	AMP +					
			1	4	5	6	7	8
Gram-positive bacteria	*S. aureus* ATCC 29213	5	**2-fold**	None	None	None	None	**2-fold**
	*S. aureus* MRSA ATCC 43300	50	**4-fold**	None	**4-fold**	**4-fold**	**4-fold**	None
	*S. epidermidis* ATCC 12228	3.125	**2-fold**	None	ND	**2-fold**	**2-fold**	None
	*E. faecalis* ATCC 29212	6.25	**2-fold**	None	ND	**2-fold**	**2-fold**	None
	*B. subtilis* ATCC 6633	0.5	**2-fold**	None	**2-fold**	**2-fold**	None	None
Gram-negative bacteria	*E. coli* ATCC 35218	>100	None	None	None	None	None	None
	*E. coli* AG100	25	**2-fold**	None	ND	**2-fold**	**2-fold**	None
	*S.* Typhimurium SEO1	6.25	None	None	**2-fold**	None	**2-fold**	None
	*K. pneumoniae* ATCC 700603	>100	None	None	None	None	None	None
	*P. aeruginosa* ATCC 27853	>100	None	None	None	None	None	None

*The bold letters indicate a decrease in the MIC of AMP; ND: not determined.

**Table 5.** Reduction of MIC values of tetracycline (TET) in combination with compounds **1**. **4–8** in bacterial strains.

Bacterial strains		MIC (µM)						
		TET	TET +					
			1	4	5	6	7	8
Gram-positive bacteria	*S. aureus* ATCC 29213	2.5	None	None	None	None	None	None
	*S. aureus* MRSA ATCC 43300	2.5	**2-fold**	None	**2-fold**	**2-fold**	**2-fold**	None
	*S. epidermidis* ATCC 12228	>100	None	None	ND	None	None	None
	*E. faecalis* ATCC 29212	100	**2-fold**	None	ND	**2-fold**	**2-fold**	None
	*B. subtilis* ATCC 6633	0.625	None	None	None	None	None	None
Gram-negative bacteria	*E. coli* ATCC 35218	6.25	2-fold increase	None	ND	None	None	None
	*E. coli* AG100	6.25	None	None	ND	None	None	None
	*S.* Typhimurium SEO1	6.25	None	None	None	None	None	None
	*K. pneumoniae* ATCC 700603	>100	None	None	None	None	None	None
	*P. aeruginosa* ATCC 27853	>100	None	None	None	None	None	None

*The bold letters indicate a decrease in the MIC of TET; ND: not determined.

The combination of naringenin (**1**), cilicione-a (**5**), rosmarinic acid (**6**), and rosmarinic acid methyl ester (**7**) with CIP increased the potency of CIP in two- to eightfold in some bacteria (Table 2). *K. pneumoniae* ATCC 700603 exhibited the highest susceptibility to naringenin (**1**), cilicione-a (**5**), rosmarinic acid (**6**), and rosmarinic acid methyl ester (**7**) combined with CIP, reducing the MIC of CIP from 3.125 to 0.39 µM (eightfold) by **1** and **7** and to 0.78 µM (fourfold) by **5** and **6**. 5,6-Dihydroxy-7,3′,4′- trimethoxyflavone (**4**) and fulgidic acid (**8**) decreased the MIC of CIP twofold only against *B. subtilis*; however, they reduced the effectiveness of CIP by half against *S.* Typhimurium SEO1, *K. pneumoniae* ATCC 700603, and *P. aeruginosa* ATCC 27853. *S.* Typhimurium SEO1 was the only strain in which all compounds, except for **5**, caused a twofold increase in the MIC of CIP.

Combination of compounds with GEN resulted in the highest MIC reduction effect of naringenin (**1**), rosmarinic acid (**6**), and rosmarinic acid methyl ester (**7**) (Table 3). In particular, **1** potentiated the effect of GEN in *S. aureus* ATCC 29213, *S. aureus* MRSA ATCC-43300, *E. faecalis* ATCC 29212, *B. subtilis* ATCC 6633, and *E. coli* AG100. Against the drug-resistant *E. coli* AG100 strain, combinations of GEN with naringenin (**1**), rosmarinic acid (**6**), and rosmarinic acid methyl ester (**7**) substantially modulated the effect of the antibiotic resulting in eightfold reductions in MICs (from 0.78 µM to 0.097 µM). In combinations with GEN and CIP, **1**, **5**, **6**, and **7** demonstrated the ability to enhance antibiotic activity against *B. subtilis* ATCC 6633.

Combinations of naringenin (**1**), rosmarinic acid (**6**), and rosmarinic acid methyl ester (**7**) with AMP improved the antibiotic effect against most of the tested bacteria by two- or fourfold (Table 4). Naringenin (**1**) enhanced the activity of AMP against all Gram-positive bacteria and against *E. coli* AG100. Notably, against the drug-resistant *S. aureus* MRSA ATCC 43300 strain naringenin (**1**), cilicione-a (**5**), rosmarinic acid (**6**), and rosmarinic acid methyl ester (**7**) were found to potentiate the activity of AMP, resulting in a reduction of the MIC from 50 to 12.5 µM.

The summer savory metabolites naringenin (**1**), cilicione-a (**5**), rosmarinic acid (**6**), and rosmarinic acid methyl ester (**7**) showed the smallest reductions in MICs when combined with TET (Table 5). These compounds enhanced the antibiotic effect only against *S. aureus* MRSA ATCC 43300 and *E. faecalis* ATCC 29212, reducing the MICs by half. In contrast, 5,6-dihydroxy-7,3′,4′- trimethoxyflavone (**4**) and fulgidic acid (**8**) markedly enhanced the activity of TET, resulting in significantly lower MIC values.

## Discussion

In traditional medicine, summer savory has been used to treat various respiratory and gastrointestinal disorders, many of which are associated with microbial infections. In recent years, numerous studies have demonstrated the antibacterial and antibiofilm activities of *S. hortensis* extracts and essential oils against a broad range of bacterial strains (Lesjak et al. 2016; Ramezani et al. 2016; Harmati et al. 2017; Masoum et al. 2018; Sharifi et al. 2018; Huwaimel et al. 2023). However, to date, no studies have investigated the interactions between the secondary metabolites of summer savory and conventional antibiotics.

### Isolation and identification of compounds

Ten compounds were isolated from the aerial parts of *S. hortensis;* these compounds belong to the group of flavonoids [naringenin (**1**), apigenin (**2**), 5,6,4′-trihydroxy-7,3′-dimethoxyflavone (**3**), 5,6-dihydroxy-7,3′,4′- trimethoxyflavone (**4**), and cilicione-a (**5**)], cinnamic acid derivatives [rosmarinic acid (**6**) and rosmarinic acid methyl ester (**7**)], oxylipins [fulgidic acid (**8**)] and monoterpene glucosides [carvacrol-*β*-*O*-glucoside (**9**)] and phenylethyl alcohols [3-hydroxytyrosol (**10**)]. The structures were determined by NMR spectroscopy and comparison with published data. Naringenin (**1**), apigenin (**2**), rosmarinic acid (**6**), and rosmarinic acid methyl ester (**7**) are widely occurring in the plant kingdom, and their NMR assignments agreed well with data published by Zhang et al. (2006), Kim et al. (2006), Akoury (2019), and Sina Içen et al. (2021), respectively. 5,6,4′-Trihydroxy-7,3′-dimethoxyflavone (**3**) and 5,6-dihydroxy-7,3′,4′-trimethoxyflavone (**4**) were described previously from various *Satureja* species (Skoula et al. 2005; Maldonado and Ortega, 1997; Malmir et al. 2012; Davoodi et al. 2018;  Nagao et al., 2002).

**Figure 2.**
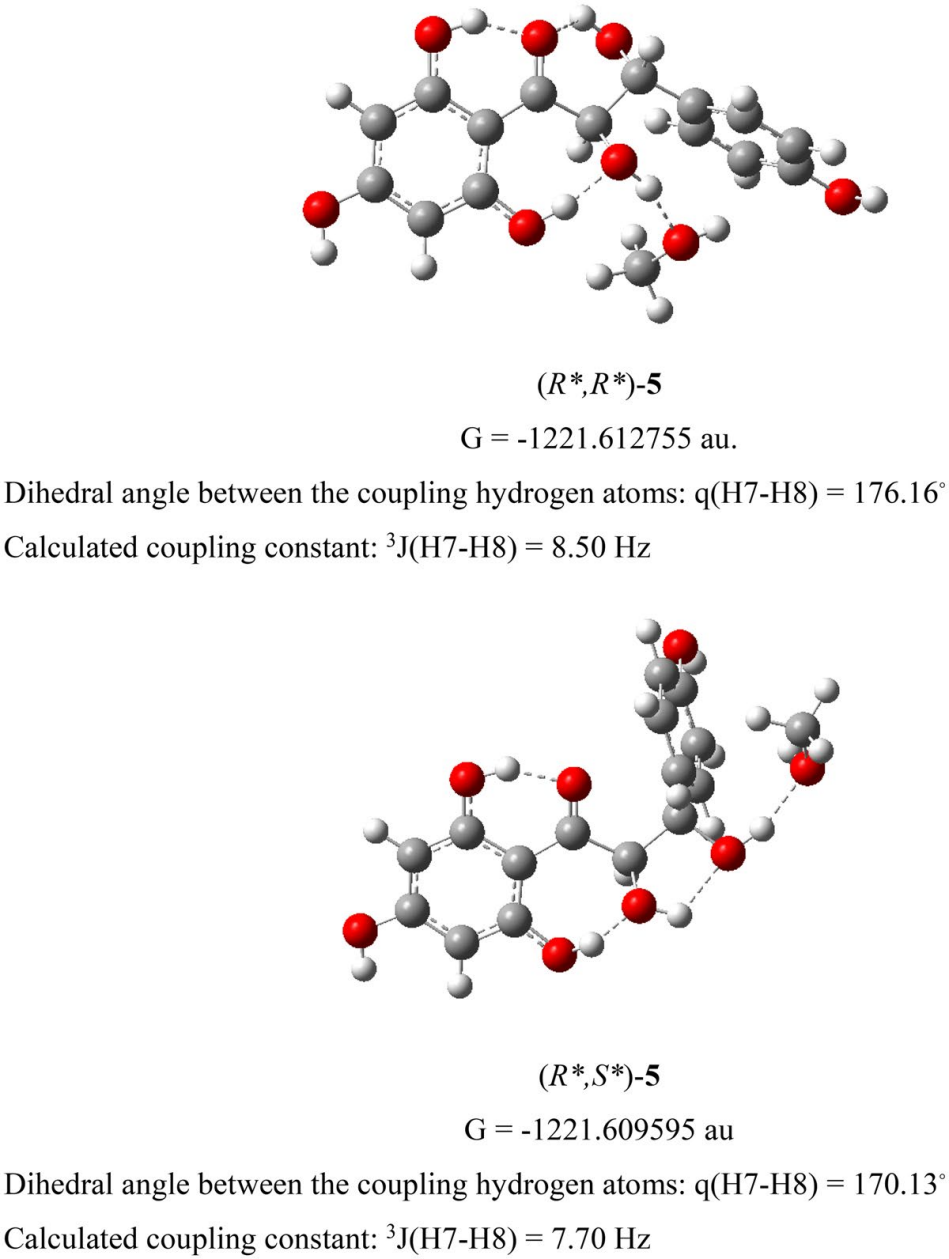
Optimized structure and gibbs free energy of measurements for cilicione-a (**5**) stereoisomers.

The dihydrochalcone derivative cilicione-a (**5**) has previously been isolated only from *Thymus cilicicus* (Ahmed and Al-Howiriny 2007), although the stereochemistry at C-7 and C-8 was not determined. To establish its relative configuration, the two possible diastereomers, (R*,R*)-**24** and (R*,S*)-**24**, were subjected to comparative DFT modeling. To better simulate the conditions of the NMR measurements, a methanol molecule was included in each model to complement the network of intramolecular hydrogen bonds, which are expected to restrict rotation around the C7–C8 bond. The calculations provided the relative thermodynamic stability of the two diastereomers, indicating that under NMR measurement conditions, (R*,R*)-**24** is more stable than (R*,S*)-**24** by 1.98 kcal/mol. Diagnostic theoretical vicinal coupling constants,^3^*J*(H7–H8), were estimated using the generalized ^3^*J*_HH_ equation (Haasnoot et al. 1980), based on the dihedral angles obtained from the optimized structures. The calculated coupling constant for (R*,R*)-**24** (8.50 Hz) was closer to the experimental value (11.6 Hz) than that of (R*,S*)-**24** (7.70 Hz) (Figure 2). Taken together, the comparison of calculated and experimental coupling constants, along with the relative thermodynamic stability, supports the assignment of the compound as (R*,R*)-**24.**

Fulgidic acid (**8**) was isolated here for the first time from the Lamiaceae family, and its NMR data agreed with data published by Zhang et al. (2024). Carvacrol-*β*-*O*-glucoside (**9**) was previously published from some Lamiaceae species: *Monarda punctata* (Yamada et al. 2010), *Thymus vulgaris* (Skopp and Hörster 1976), *Lavandula multiflora* (Sosa et al. 2005), and *Origanum vulgare* (Milos et al. 2000). However, this is the first report of its isolation from *Satureja*. The aglycon carvacrol is one of the main compounds of the essential oil of *S. hortensis* (Lesjak et al. 2016).

3-Hydroxytyrosol (**10**), a typical compound of olive leaves and fruits (Kalampaliki et al. 2019), was also identified from summer savory. To our knowledge, this is the first report of **10**, **3**, and **7** isolated from *S. hortensis*. Our 2D NMR studies allowed the assignment of NMR signals in solvents other than those previously reported for **3**, **8**, and **9** (Materials and methods).

### Interactions of the isolated compounds with antibiotics

The interactions of conventional antibiotics—CIP, GEN, AMP, and TET—with six compounds (**1**, **4**–**8**) isolated from *S. hortensis* were investigated. Among these, compounds **1** and **6** are widely distributed in the plant kingdom. Apigenin (**2**) was not included in the present study, as its interaction with antibiotics was previously evaluated under identical experimental conditions.

Prior to assessing the interactions, an initial antibacterial screening was performed against five Gram-positive and five Gram-negative bacterial strains to determine the MICs of the tested antibiotics and isolated compounds. None of the compounds exhibited antibacterial activity at concentrations up to 200 µM (MIC > 200 µM).

Among the tested combinations, *B. subtilis* ATCC 6633 exhibited the highest sensitivity, whereas *S. enterica* serovar Typhimurium SEO1 showed the lowest sensitivity to the compounds when combined with CIP, GEN, and AMP.

Naringenin (**1**) exhibited the strongest potentiating effect, enhancing the activities of CIP, GEN, and AMP. These combinations reduced the MICs by eightfold against *K. pneumoniae* (with CIP) and drug-resistant *E. coli* AG100 (with GEN), and by fourfold against *S. aureus* ATCC 29213 (with GEN), *S. aureus* MRSA ATCC 43300 (with GEN and AMP), and *B. subtilis* ATCC 6633 (with GEN). These results align with previous reports demonstrating the potentiating effects of naringenin (**1**). For example, Yi et al. (2024) reported an eightfold reduction in the MIC of amikacin against multidrug-resistant *E. coli* when combined with **1**. Similarly, Mohammed et al. (2015) showed that **1** significantly decreased the MIC of CIP against a clinical MRSA isolate. Duda-Madej et al. (2022) also described a synergistic interaction between naringenin (**1**) and oxacillin against MRSA, while Ng’uni et al. (2015) observed synergism with AMP, TET, methicillin, and vancomycin against *S. aureus* and MRSA. Notably, the present study is the first to demonstrate the adjuvant potential of naringenin (**1**) in combination with antibiotics against *E. faecalis* ATCC 29212, *B. subtilis* ATCC 6633, *K. pneumoniae* ATCC 700603, and *P. aeruginosa* ATCC 27853.

Both rosmarinic acid (**6**) and its methyl ester (**7**) exhibited promising activity in combination assays, each reducing the MICs of CIP, GEN, AMP, and TET against four Gram-positive and four Gram-negative bacterial strains. Notably, a fourfold and eightfold potentiation was observed against the drug-resistant strains *S. aureus* MRSA ATCC 43300 and *E. coli* AG100 when combined with AMP and GEN, respectively. Rosmarinic acid methyl ester (**7**) demonstrated slightly greater potency than rosmarinic acid (**6**), as evidenced by a twofold MIC reduction of CIP against *E. faecalis* ATCC 29212 and of AMP against *Salmonella* Typhimurium SEO1, as well as a more pronounced reduction in the MIC of CIP against *K. pneumoniae* ATCC 700603. Although previous studies have reported synergistic effects of rosmarinic acid (**6**) in combination with antibiotics such as vancomycin, ofloxacin, and amoxicillin against *S. aureus* (Ekambaram et al. 2016), these interactions were not observed in the present study. Zhang et al. (2024) also demonstrated synergy between **6** and the cephalosporin ceftiofur in anti-MRSA activity. Therefore, the potentiating effects observed for **6** and **7** in this study represent novel findings.

Among the tested compounds, fulgidic acid (**8**) was the only one featuring an aliphatic chain structure. Likely due to the absence of a phenolic moiety, it exhibited only a weak ability to potentiate antibiotic activity. A twofold reduction in the MICs was observed in combination with CIP, AMP, and GEN against *S. aureus* ATCC 29213 (with AMP and GEN), *B. subtilis* ATCC 6633 (with GEN and CIP), and *K. pneumoniae* ATCC 700603 (with GEN).

When comparing the effectiveness of naringenin (**1**) and apigenin (**2**), which differ only by the presence of a C-2/C-3 double bond, both exhibited similar potentiating effects in combination with CIP against *S. aureus* ATCC 29213 and *S. aureus* MRSA 43300. However, apigenin (**2**) showed no substantial influence on antibiotic susceptibility in other strains, particularly Gram-negative bacteria (Kincses et al. 2024). 5,6-Dihydroxy-7,3′,4′-trimethoxyflavone (**4**), which also possesses a flavone backbone like **2**, demonstrated similarly limited activity compared with naringenin (**1**), with its most notable effect being a fourfold MIC reduction of *B. subtilis* ATCC 6633 when combined with GEN.

Our assays provided insights into the antibiotic adjuvant potential of cilicione-a (**5**), a dihydrochalcone derivative. While previous studies have demonstrated that both natural and synthetic chalcones can significantly enhance the activity of various antibiotics (Lee et al. 2010; Tran et al. 2012; Božić et al. 2014), these investigations were limited to classical chalcones. In contrast, compound **5** is a 7,8-dihydro-7,8-dihydroxychalcone, representing a structurally distinct scaffold. Notably, **5** exhibited its most pronounced potentiating effect in combination with CIP against *K. pneumoniae* and with AMP against MRSA, each resulting in a fourfold reduction in MIC.

In certain cases, combinations of compounds **1** and **4**–**8** with CIP, GEN, and TET against the tested Gram-negative bacterial strains resulted in a twofold increase in MICs. Such increases in MIC are of particular concern in drug discovery and clinical therapy, as it may lead to reduced treatment efficacy, the requirement for higher antibiotic doses, or prolonged infections. Although clinically significant antagonistic interactions have been reported only infrequently, some examples include combinations of *β*-lactam and macrolide antibiotics with other bacteriostatic agents (Acar 2000).

The observed enhancement of antibiotic activity by the investigated compounds may arise through several mechanisms. One common mechanism is the increased permeability of the bacterial cell envelope, which facilitates antibiotic uptake. In other instances, direct physical interactions at the target site can enhance antibacterial activity. More complex synergy may involve alterations in bacterial physiology that indirectly improve antibiotic efficacy (Bollenbach 2015). For example, rosmarinic acid (**6**), has been shown to inhibit bacterial surface proteins, potentially impairing the ability of pathogens to evade antibiotic action. In MRSA and *S. aureus*, co-administration of **6** with a glycopeptide antibiotic significantly reduced the expression of microbial surface components recognizing adhesive matrix molecules compared with rosmarinic acid (**6**) alone (Ekambaram et al. 2016). Naringenin (**1**) exerts antimicrobial effects on MRSA strains by decreasing membrane fluidity in both hydrophilic and hydrophobic regions of the bacterial membranes (Duda-Madej et al. 2022).

## Conclusions

This study demonstrated that *S. hortensis* is a valuable source of secondary metabolites (**1**–**10**) with the potential to enhance the efficacy of conventional antibiotics. Although six of the isolated compounds (**1**, **4**–**8**) exhibited no intrinsic antibacterial activity at concentrations up to 200 µM, their use at sub-inhibitory levels significantly potentiated the activity of CIP, GEN, AMP, and TET against both Gram-positive and Gram-negative bacterial strains, including drug-resistant variants. The observed potentiating effects are likely mediated through multiple mechanisms, including alterations in membrane structure and permeability, inhibition of adhesion-related protein expression, and interference with key metabolic pathways. These findings underscore the potential of natural product–antibiotic combinations to improve therapeutic outcomes, reduce required antibiotic doses, and support strategies aimed at combating antimicrobial resistance.

## Data Availability

The datasets used and/or analyzed are available from the corresponding author upon reasonable request.

## References

[CIT0001] Acar JF. 2000. Antibiotic synergy and antagonism. Med Clin North Am. 84(6):1391–1406. 10.1016/S0025-7125(05)70294-7 11155849

[CIT0002] Ahmed B, Al-Howiriny TA. 2007. Two new hydroxy chalcone derivatives from *Thymus cilicicus*. Z. Naturfoesch B. 62(1):121–124. 10.1515/znb-2007-0118

[CIT0003] Akoury E. 2019. Isolation and structural elucidation of rosmarinic acid by nuclear magnetic resonance spectroscopy. Am Res J Chem. 1(1):17–23. 10.21694/2577-5898.17003

[CIT0004] Ayaz M et al. 2019. Synergistic interactions of phytochemicals with antimicrobial agents: potential strategy to counteract drug resistance. Chem Biol Interact. 308:294–303. 10.1016/j.cbi.2019.05.050 31158333

[CIT0005] Bollenbach T. 2015. Antimicrobial interactions: mechanisms and implications for drug discovery and resistance evolution. Curr Opin Microbiol. 27:1–9. 10.1016/j.mib.2015.05.008 26042389

[CIT0006] Božić DD, Milenković M, Ivković B, Ćirković I. 2014. Antibacterial activity of three newly-synthesized chalcones & synergism with antibiotics against clinical isolates of methicillin-resistant *Staphylococcus aureus*. Indian J Med Res. 140(1):130–137. 25222788 PMC4181146

[CIT0007] Davoodi M, Rustaiyan A, Ebrahimi SN. 2018. Monoterpene flavonoid from aerial parts of *Satureja khuzistanica*. RecNatProd. 12(2):175–178. 10.25135/rnp.19.17.06.109

[CIT0008] Dhanda G, Acharya Y, Haldar J. 2023. Antibiotic adjuvants: a versatile approach to combat antibiotic resistance. ACS Omega. 8(12):10757–10783. 10.1021/acsomega.3c00312 37008128 PMC10061514

[CIT0009] Duda-Madej A, Stecko J, Sobieraj J, Szymańska N, Kozłowska J. 2022. Naringenin and its derivatives—Health-promoting phytobiotic against resistant bacteria and fungi in humans. Antibiotics. 11(11):1628. 10.3390/antibiotics11111628 36421272 PMC9686724

[CIT0010] Ejaz A et al. 2023. A comprehensive review of summer savory (*Satureja hortensis* L.): promising ingredient for production of functional foods. Front Pharmacol. 14:1198970. 10.3389/fphar.2023.1198970 37554989 PMC10406440

[CIT0011] Ekambaram SP et al. 2016. Antibacterial synergy between rosmarinic acid and antibiotics against methicillin-resistant *Staphylococcus aureus*. J Intercult Ethnopharmacol. 5(4):358–363. 10.5455/jice.20160906035020 27757265 PMC5061478

[CIT0012] Getahun YA, Ali DA, Taye BW, Alemayehu YA. 2022. Multidrug-resistant microbial therapy using antimicrobial peptides and the CRISPR/Cas9 system. Vet Med (Auckl). 13:173–190. 10.2147/VMRR.S366533 35983086 PMC9379109

[CIT0013] Haasnoot CAG, DeLeeuw FAAM, Altona C. 1980. The relationship between proton-proton NMR coupling constants and substituent electronegativities—I: an empirical generalization of the karplus equation. Tetrahedron. 36(19):2783–2792. 10.1016/0040-4020(80)80155-4

[CIT0014] Harmati M et al. 2017. Binary mixture of *Satureja hortensis* and *Origanum vulgare* subsp. *hirtum* essential oils: *in vivo* therapeutic efficiency against *Helicobacter pylori* infection. Helicobacter. 22(2):e12350. 10.1111/hel.12350 27578489

[CIT0015] Hehre JW, Radom L, Schleyer PVR, Pople JA. 1986. Ab initio molecular orbital theory. New York: Wiley.

[CIT0017] Huwaimel B et al. 2023. Novel landmarks on the journey from natural products to pharmaceutical formulations: phytochemical, biological, toxicological and computational activities of *Satureja hortensis* L. Food Chem Toxicol. 179:113969. 10.1016/j.fct.2023.113969 37517548

[CIT0018] Kalampaliki AD, Giannouli V, Skaltsounis AL, Kostakis IK. 2019. A three-step, Gram-scale synthesis of hydroxytyrosol, hydroxytyrosol acetate, and 3,4-dihydroxyphenylglycol. Molecules. 24(18):3239. 10.3390/molecules24183239 31492013 PMC6767028

[CIT0019] Kim JS et al. 2006. Chemical constituents of the root of *Dystaenia takeshimana* and their anti-inflammatory activity. Arch Pharm Res. 29(8):617–623. 10.1007/BF02968244 16964755

[CIT0020] Kincses A, Ghazal TSA, Hohmann J. 2024. Synergistic effect of phenylpropanoids and flavonoids with antibiotics against Gram-positive and Gram-negative bacterial strains. Pharm Biol. 62(1):659–665. 10.1080/13880209.2024.2389105 39126171 PMC11318484

[CIT0021] Lee GS et al. 2010. Antibacterial and synergistic activity of prenylated chalcone isolated from the roots of *Sophora flavescens*. J Korean Soc Appl Biol Chem. 53(3):290–296. 10.3839/jksabc.2010.045

[CIT0022] Lesjak M et al. 2016. Binary and tertiary mixtures of *Satureja hortensis* and *Origanum vulgare* essential oils as potent antimicrobial agents against *Helicobacter pylori*. Phytother Res. 30(3):476–484. 10.1002/ptr.5552 26686190

[CIT0023] Maldonado E, Ortega A. 1997. Neo-clerodane diterpenes from *Salvia thymoides*. Phytochemistry. 46(7):1249–1254. 10.1016/S0031-9422(97)80021-0

[CIT0024] Malmir M, Gohari AR, Saeidnia S. 2012. Flavonoids from the aerial parts of *Satureja khuzestanica*. Planta Med. 78(11):1226. 10.1055/s-0032-1321052

[CIT0025] Masoum S, Samadi N, Mehrara B, Mahboubi M. 2018. Potentiality of independent component regression in assessment of the peaks responsible for antimicrobial activity of *Satureja hortensis* L. and *Oliveria decumbens* Vent. using GC–MS. J Iran Chem Soc. 15(9):2007–2016. 10.1007/s13738-018-1398-8

[CIT0026] Mastelić J, Jerković I, Vinković M, Džolić Z, Vikić-Topić D. 2004. Synthesis of selected naturally occurring glucosides of volatile compounds. Their chromatographic and spectroscopic properties. Croat Chem Acta. 77(3):491–500.

[CIT0027] Matsunaga N, Hayakawa K. 2018. Estimating the impact of antimicrobial resistance. Lancet Glob Health. 6(9):e934–e935. 10.1016/S2214-109X(18)30325-5 30103983

[CIT0028] Milos M, Mastelic J, Jerkovic I. 2000. Chemical composition and antioxidant effect of glycosidically bound volatile compounds from oregano (*Origanum vulgare* L. ssp. *hirtum*). Food Chem. 71(1):79–83. 10.1016/S0308-8146(00)00144-8

[CIT0029] Mohammed NH, Mostafa MI, Al-Taher AY. 2015. Augmentation effects of novel naringenin analogues and ciprofloxacin as inhibitors for nora efflux pump (EPIs) and pyruvate kinase (PK) against MRSA. J. Anim Vet Adv. 1413:386–392. (–24):

[CIT0030] Nagao T, Abe F, Kinjo J, Okabe H. 2002. Antiproliferative constituents in plants 10. Flavones from the leaves of *Lantana montevidensis* BRIQ. and consideration of structure–activity relationship. Biol Pharm Bull. 25(7):875–879. 10.1248/bpb.25.875 12132661

[CIT0031] Ng’uni T, Mothlalamme T, Daniels R, Klaasen J, Fielding BC. 2015. Additive antibacterial activity of naringenin and antibiotic combinations against multidrug resistant *Staphylococcus aureus*. Afr J Microbiol Res. 9(23):1513–1518. 10.5897/AJMR2015.7514

[CIT0032] Ramezani M, Ehtesham-Gharaee M, Khazaie M, Behravan J. 2016. *Satureja hortensis* L. methanolic extract and essential oil exhibit antitumor activity. J Essent Oil Bear Pl. 19(1):148–154. 10.1080/0972060X.2015.1060872

[CIT0033] Sharifi A, Mohammadzadeh A, Zahraei Salehi T, Mahmoodi P. 2018. Antibacterial, antibiofilm and antiquorum sensing effects of *Thymus daenensis* and *Satureja hortensis* essential oils against *Staphylococcus aureus* isolates. J Appl Microbiol. 124(2):379–388. 10.1111/jam.13639 29144601

[CIT0034] Sina Içen M, Gürbüz İ, Bedir E, Günbatan T, Demirci F. 2021. Isolation of rosmarinic acid and methyl rosmarinate as lipoxygenase inhibitors from *Salvia palaestina* Benth. by activity-guided fractionation. S Afr J Bot. 141:177–182. 10.1016/j.sajb.2021.04.030

[CIT0035] Skopp K, Hörster H. 1976. Sugar bound regular Monoterpenes, Part I Thymol– and Carvacrolglykosides in *Thymus vulgaris*. Planta Med. 29(3):208–215. 10.1055/s-0028-1097653 133368

[CIT0036] Skoula M, Grayer RJ, Kite GC. 2005. Surface flavonoids in *Satureja thymbra* and *Satureja spinosa* (Lamiaceae). Biochem Syst Ecol. 33(5):541–544. 10.1016/j.bse.2004.10.003

[CIT0037] Sosa S et al. 2005. Extracts and constituents of *Lavandula multifida* with topical anti-inflammatory activity. Phytomedicine. 12(4):271–277. 10.1016/j.phymed.2004.02.007 15898704

[CIT0038] Tomasi J, Mennucci B, Cancès E. 1999. The IEF version of the PCM solvation method: an overview of a new method addressed to study molecular solutes at the QM ab initio level. J Mol Struct THEOCHEM. 464(1-3):211–226. 10.1016/S0166-1280(98)00553-3

[CIT0039] Tran TD et al. 2012. Synthesis and anti Methicillin resistant *Staphylococcus aureus* activity of substituted chalcones alone and in combination with non-beta-lactam antibiotics. Bioorg Med Chem Lett. 22(14):4555–4560. 10.1016/j.bmcl.2012.05.112 22727643

[CIT0041] Yamada K, Murata T, Kobayashi K, Miyase T, Yoshizaki F. 2010. A lipase inhibitor monoterpene and monoterpene glycosides from *Monarda punctata*. Phytochemistry. 71(16):1884–1891. 10.1016/j.phytochem.2010.08.009 20832830

[CIT0042] Yi L et al. 2024. In vitro antimicrobial synergistic activity and the mechanism of the combination of naringenin and amikacin against antibiotic-resistant *Escherichia coli*. Microorganisms. 12(9):1871. 10.3390/microorganisms12091871 39338545 PMC11433787

[CIT0043] Zhang P et al. 2024. Potential anti-inflammatory constituents from *Aesculus wilsonii* seeds. Molecules. 29(5):1136. 10.3390/molecules29051136 38474647 PMC10934013

[CIT0044] Zhang X et al. 2006. Anti-inflammatory activity of flavonoids from *Populus davidiana*. Arch Pharm Res. 29(12):1102–1108. 10.1007/BF02969299 17225458

[CIT0045] Zhang Z et al. 2024. Rosmarinic acid restores the ceftiofur antibacterial activity against methicillin-resistant *Staphylococcus aureus* by inhibiting sortase A. J Agric Food Chem. 72(49):27215–27224. 10.1021/acs.jafc.4c07505 39614811

[CIT0046] Zhao Y, Truhlar DG. 2008. The M06 suite of density functionals for main group thermochemistry, thermochemical kinetics, noncovalent interactions, excited states, and transition elements: two new functionals and systematic testing of four M06-class functionals and 12 other functionals. Theor Chem Account. 120(1-3):215–241. 10.1007/s00214-007-0310-x

